# Combined magnetic resonance imaging and serum analysis reveals distinct multiple sclerosis types

**DOI:** 10.1093/brain/awaf331

**Published:** 2025-12-02

**Authors:** Charles Willard, Lemuel Puglisi, Daniele Ravi, Mariia Dmitrieva, Rozemarijn M Mattiesing, Frederik Barkhof, Daniel C Alexander, Danielle E Harlow, Daniela Piani-Meier, Arman Eshaghi

**Affiliations:** Department of Research and Analysis, Queen Square Analytics Limited, London EC1V 2NX, UK; Department of Research and Analysis, Queen Square Analytics Limited, London EC1V 2NX, UK; Department of Mathematics and Computer Science, University of Catania, Catania 95124, Italy; MIFT Department, University of Messina, Messina 98122, Italy; Department of Research and Analysis, Queen Square Analytics Limited, London EC1V 2NX, UK; MS Center Amsterdam, Radiology and Nuclear Medicine, Vrije Universiteit Amsterdam, Amsterdam Neuroscience, Amsterdam UMC, Amsterdam, The Netherlands; Department of Research and Analysis, Queen Square Analytics Limited, London EC1V 2NX, UK; Department of Medical Physics and Biomedical Engineering, University College London, London WC1E 6BT, UK; Department of Radiology and Nuclear Medicine, Neuroscience Campus Amsterdam, VU University Medical Center, Amsterdam 1081 BT, The Netherlands; Queen Square Multiple Sclerosis Centre, Department of Neuroinflammation, Queen Square Institute of Neurology, Faculty of Brain Sciences, University College London, London WC1N 3BG, UK; Department of Brain Repair and Rehabilitation, Queen Square Institute of Neurology, University College London, London WC1N 3BG, UK; UCL Hawkes Institute, University College London, London WC1V 6LJ, UK; Department of Research and Analysis, Queen Square Analytics Limited, London EC1V 2NX, UK; UCL Hawkes Institute, University College London, London WC1V 6LJ, UK; Department of Computer Science, University College London, London WC1E 6EA, UK; Neurology & Immunology Medical Unit, EMD Serono Research & Development Institute, Inc., Billerica, MA 01821, USA, an affiliate of Merck KGaA, Darmstadt, Germany; Neurology & Immunology Medical Unit, Ares Trading SA, Eysins 1262, Switzerland, an affiliate of Merck KGaA, Darmstadt, Germany; Department of Research and Analysis, Queen Square Analytics Limited, London EC1V 2NX, UK; Department of Medical Physics and Biomedical Engineering, University College London, London WC1E 6BT, UK; Queen Square Multiple Sclerosis Centre, Department of Neuroinflammation, Queen Square Institute of Neurology, Faculty of Brain Sciences, University College London, London WC1N 3BG, UK; UCL Hawkes Institute, University College London, London WC1V 6LJ, UK; Department of Computer Science, University College London, London WC1E 6EA, UK

**Keywords:** disease phenotyping, machine learning, precision medicine, neurodegeneration, neuroinflammation

## Abstract

Multiple sclerosis (MS) is a highly heterogeneous disease in its clinical manifestation and progression. Predicting individual disease courses is key for aligning treatments with underlying pathobiology. We developed an unsupervised machine learning model integrating MRI-derived measures with serum neurofilament light chain (sNfL) levels to identify biologically informed MS subtypes and stages. Using a training cohort of patients with relapsing–remitting and secondary progressive MS (*n* = 189), with validation on a newly diagnosed population (*n* = 445), we discovered two distinct subtypes defined by the timing of sNfL elevation and MRI abnormalities (early- and late-sNfL types).

In comparison to MRI-only models, incorporating sNfL with MRI improved correlations of data-derived stages with the Expanded Disability Status Scale in the training (Spearman’s ρ = 0.420 versus MRI-only ρ = 0.231, *P* = 0.001) and external test sets (ρ = 0.163 for MRI–sNfL, versus ρ = 0.067 for MRI-only). The early-sNfL subtype showed elevated sNfL, corpus callosum injury and early lesion accrual, reflecting more active inflammation and neurodegeneration, whereas the late-sNfL group showed early volume loss in the cortical and deep grey matter volumes, with later sNfL elevation. Cross-sectional subtyping predicted longitudinal radiological activity: the early-sNfL group showed a 144% increased risk of new lesion formation (hazard ratio = 2.44, 95% confidence interval 1.38–4.30, *P* < 0.005) compared with the late-sNfL group. Baseline subtyping, over time, predicted treatment effect on new lesion formation on the external test set (faster lesion accrual in early-sNfL compared with late-sNfL, *P* = 0.01), in addition to treatment effects on brain atrophy (early sNfL average percentage brain volume change: −0.41, late-sNfL = −0.31, *P* = 0.04).

Integration of sNfL provides an improved framework in comparison to MRI-only subtyping of MS to stage disease progression and inform prognosis. Our model predicted treatment responsiveness in early, more active disease states. This approach offers a powerful alternative to conventional clinical phenotypes and supports future efforts to refine prognostication and guide personalized therapy in MS.

## Teaser

Willard & Puglisi *et al.* used MRI and serum neurofilament light chain (sNfL) to define two subtypes of multiple sclerosis, based on the timing of sNfL elevation and MRI abnormalities. Their model outperformed an MRI-only model in predicting disability, disease activity, and treatment response, offering a new path to personalised care.

**See Brummer and Fleischer (https://doi.org/10.1093/brain/awaf400) for a scientific commentary on this article.**

## Introduction

Multiple sclerosis (MS) affects >2.8 million people globally.^1^ Although clinical descriptors categorize MS into relapsing–remitting, secondary progressive and primary progressive, emerging evidence reveals that these fail to capture the underlying biological continuum.^2,3^ Although distinct in their disease burden, these clinical phenotypes share pathobiological mechanisms.^4,5^ As a result, our capacity to predict its course and personalize treatment remains limited by our reliance on traditional clinical descriptors. A shift towards a subtyping system grounded in pathobiological underpinnings promises to facilitate earlier, more targeted therapeutic interventions, potentially even before symptom onset, improving our ability to predict individual patient outcomes and personalize treatment strategies.

Unsupervised machine learning offers an objective way to uncover MS types based on underlying disease biology (pathobiology) rather than relying on predefined diagnostic labels or observable symptoms. For example, Gross *et al*.^6^ identified three distinct data-derived MS types based on different immune cell markers of CD4 and CD8 T cells, natural killer cells and cytokine compartments. These data-derived subtypes showed subtle variations in disease course and treatment responses. However, their immediate clinical use is limited because specialized immune cell markers defined these subtypes, which are not collected routinely in clinical practice.

MRI provides detailed spatial information about structural damage and lesion formation, capturing the localized effects of the disease. The unsupervised learning model Subtype and Staging Inference (SuStaIn^7^) was used previously by Eshaghi *et al*.^8^ on brain MRI data from 9390 MS patients to identify three distinct MRI-derived subtypes. SuStaIn clusters patients with similar progression patterns, characterizing subtypes by the alternative order in which key variables reach thresholds of abnormality. They found a ‘lesion-led’ subtype that was more responsive to high-efficacy treatments in clinical trials and had faster worsening of disability than the other two subtypes.^8^ However, MRI alone does not fully capture the underlying neuro-axonal damage that drives disease progression. MRI is non-specific for underlying pathology and can miss subtle or ongoing neurodegeneration.^9^ Integrating widely available fluid biomarkers indicative of early and ongoing neuronal injury has the potential for improved separation of patient subgroups and enhanced precision in disease course prediction and personalized treatment selection.^10^

The serum neurofilament light chain (sNfL) level is a systemic measure of ongoing neuronal injury, reflecting disease activity and neuronal loss.^11,12^ It is a component of the neuronal cytoskeleton and, therefore, a sensitive indicator of neuro-axonal damage. In MS, the sNfL level increases at disease onset,^13^ is associated with MRI changes of disease activity and neurodegeneration^14^ and is sensitive to treatment effects.^15,16^ Serum NfL is becoming increasingly available and accessible in clinical practice.^17,18^ Yet, because serum-based biomarkers alone still face variability, low sensitivity to disease activity and imperfect assay standardization, incorporating complementary data (such as MRI metrics) can strengthen their clinical utility.^19-21^

We hypothesized that integrating sNfL with MRI would yield biologically distinct MS subtypes with improved prognostic accuracy in comparison to MRI alone. In this study, using two independent cohorts, we aimed to: (i) develop and validate a combined MRI–sNfL subtyping system using unsupervised machine learning (SuStaIn); (ii) assess how incorporating sNfL can simplify and improve data-derived subtypes in comparison to using MRI alone; and (iii) evaluate how these novel subtypes evolve in their brain atrophy, treatment response and new lesion development.

## Materials and methods

### Overview

**Figure 1:**
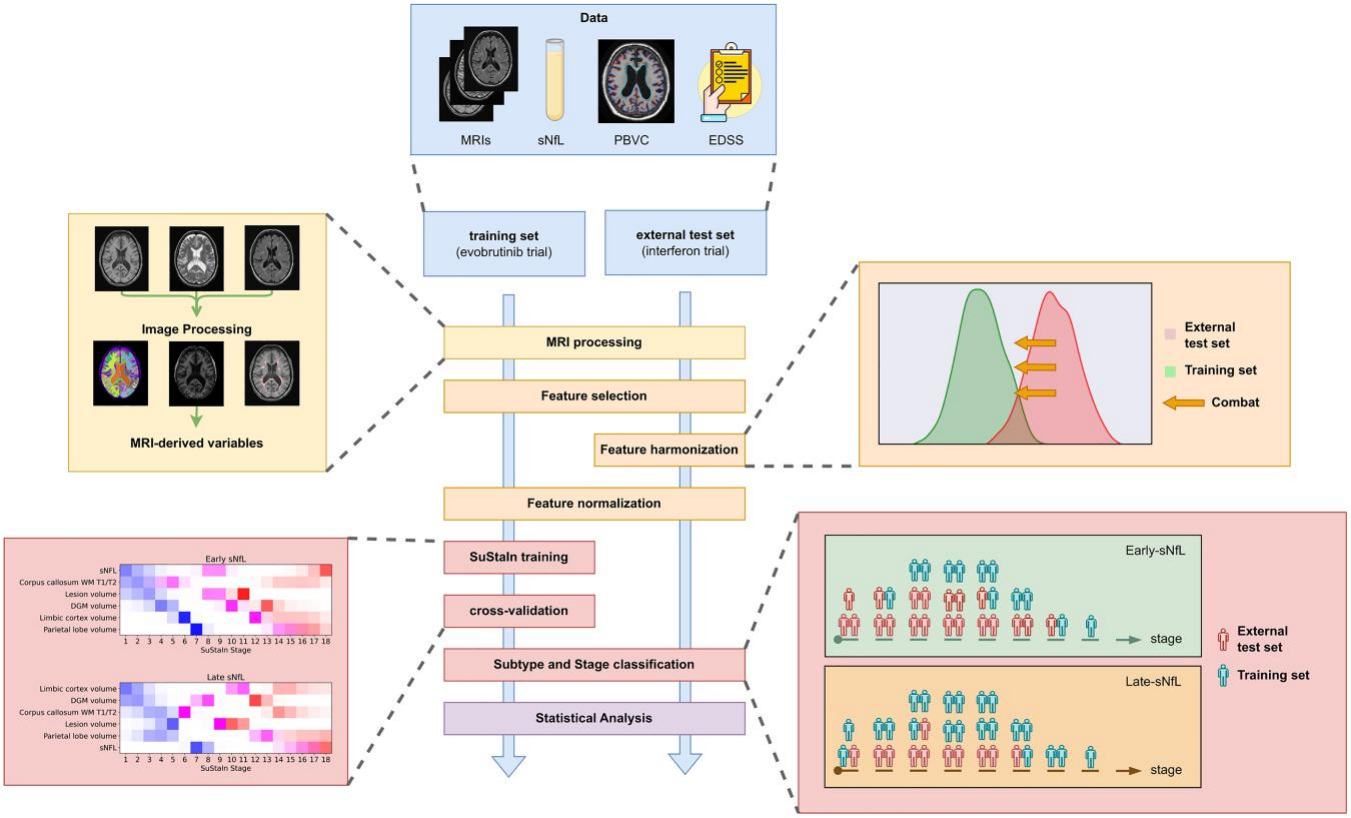
**Overview of the study.** Initially, we processed MRIs to derive 35 features from 17 targeted brain regions, white matter hyperintensity volumes and T1/T2 ratios of white matter regions. Next, we selected five MRI features within SuStaIn based on their correlation with EDSS. We harmonized the features from the external test dataset to match the distribution of the features in the training set. We normalized the features by computing *z*-scores relative to a healthy population. We trained a SuStaIn model with the selected MRI features and sNfL measures and performed cross-validation to select the optimal model. We applied the trained SuStaIn model to cross-sectional and longitudinal data from subjects in the training and external test datasets. We performed a statistical analysis to investigate how the newly obtained subtyping and staging system is related to clinical variables, and to compare our models with an existing MRI-based SuStaIn model. EDSS = Expanded Disability Status Scale; sNfL = serum neurofilament light (chain); SuStaIn = subtype and stage inference.

Figure 1 provides an overview of our study, a retrospective analysis of two previously published longitudinal datasets. We trained an unsupervised SuStaIn model using cross-sectional MRI data and sNfL levels from a phase 2 clinical trial (referred to as training data in this manuscript).^22^ The training of this model included a feature selection step to select MRI-derived variables based on their correlation with Expanded Disability Status Scale (EDSS), which makes the pipeline not entirely unsupervised despite using an unsupervised model. Through cross-validation, we determined the optimal number and pattern of data-derived subtypes, and the most likely sequence of progression of abnormality across selected MRI variables and sNfL levels. In the SuStaIn framework, subtypes are modelled as data-driven sequences of biomarkers. Each subtype captures a distinct ordering of disease events, but given that individuals are assigned to subtypes probabilistically, from this perspective, the subtypes can be considered a continuum. The trained model assigned each patient both a stage (indicating their position along the disease progression sequence) and a subtype classification with an associated probability of subtype membership. We applied this classification to cross-sectional and longitudinal observations within the training data and an independent external interferon beta-1a trial dataset (referred to as the external test set in this manuscript).^23^ We compared our data-derived subtypes with MRI-only models to assess the added value of incorporating sNfL with MRI variables, where our approach differs only by the addition of the sNfL biomarker, reduced MRI biomarker selection and the use of harmonization on the test set. We performed statistical analyses to evaluate the relationship between clinical outcomes and the derived stages, to validate model-derived subtypes and stages against disability measures and to explore differences in outcomes across subtypes.

### Participants

We included 634 participants across training and external test datasets. For training, we used 189 participants from the previously published phase 2 clinical trial of evobrutinib,^22^ and for independent testing, we used 445 from the phase 3 clinical trial on interferon beta-1a (which we refer to as ‘early’ MS in this manuscript, although in the published manuscript,^23^ according to the now obsolete McDonald 2005 diagnostic criteria, they were referred to as clinically isolated syndrome; subsequent *post hoc* analyses of the REFLEX clinical trial using retrospective application of the McDonald 2017 criteria^24^ confirmed that almost half would have been confirmed as having MS at baseline).^23,25^ Those in the training dataset (evobrutinib trial) had established relapse-onset MS (relapsing–remitting and secondary progressive). Given that SuStaIn requires data covering the full disease course for training, we selected the evobrutinib trial data as the training set and the interferon trial data with patients at the time of diagnosis of MS and clinically isolated syndrome for the external test dataset.

In the training dataset (evobrutinib trial), we had follow-up visits at Months 3, 4, 5, 6 and 12. A total of 167 participants had sNfL measurements at screening visits. We consolidated participants into treatment and control groups based on previously reported efficacy. Those who received either placebo (*n* = 50) or evobrutinib 25 mg once daily (*n* = 46) formed our control group, because these doses had shown no significant treatment effect. Participants who received higher doses [evobrutinib 75 mg once daily (*n* = 46) or twice daily (*n* = 47)] formed our treatment group.

For the external test dataset (interferon trial), follow-up visits occurred every 3 months from baseline to Month 24 during the REFLEX trial,^23^ and every 6 months from Month 24 to Month 60 during the REFLEXION extension study,^26^ in which the control group switched to treatment. A total of 435 participants had sNfL measurements at screening. Participants were grouped based on treatment assignment: the placebo arm (*n* = 144) served as our control group, whereas those receiving either weekly (*n* = 152) or thrice weekly (*n* = 149) interferon beta-1a formed the treatment group. In our analyses, we incorporated the full 60 months of data for the treatment group but restricted control group analyses to the first 24 months, prior to crossover treatment in the extension phase.

### Ethics and consent

The clinical trial protocol and ethics approval were acquired by the institutional ethical review boards, and informed written consent was obtained from all participants or their designated caregivers (ClinicalTrials.gov IDs: NCT02975349^22^ and NCT00404352^23^).

### Assessments

#### Clinical assessments

We used EDSS assessments with data available at Months 0 (screening), 3 and 6 in the training dataset. In the external test dataset, EDSS assessments were available from Month 0 (screening) to Month 24 at 3 month intervals, and from Month 24 to Month 60 at 6 month intervals. We did not analyse relapses because they were too rare (11 of 189 subjects in the training set and 21 of 445 in the external test sets experienced a relapse).

#### sNfL measurements

The sNfL measurements for the evobrutinib phase 2 trial were analysed by Monogram Biosciences (LabCorp), using the Simoa assay.^11^ The sNfL measurements for the interferon trial were analysed by the SMSC Data Center in Basel using the Simoa assay. We analysed the laboratory-standardized *z*-scores, which were corrected for age against a control population for the evobrutinib study, and corrected for age and body mass index for the interferon study.^27^

#### MRI and acquisition protocols

We used MRIs from visits conducted at Months 0 (screening), 3, 4, 5, 6 and 12 in the training dataset. For the external test dataset, MRI scans were available at Months 0 (screening), 12, 24, 36, 48 and 60. In the training set, MRI scans were acquired using 1.5 T scanners and included two-dimensional T1-weighted, T2-weighted and fluid-attenuated inversion recovery (FLAIR) sequences. Likewise, MRI scans from the interferon trial were acquired using 1.5 T scanners but included two-dimensional T1-weighted, T2-weighted and proton density (PD) sequences. Full details of the MRI schedules and trial protocols are published elsewhere (as previously mentioned, NCT02975349^22^ and NCT00404352^23^).

### MRI processing

The objective of the MRI processing was to quantify: (i) T2-hyperintense white matter lesion volumes; (ii) volumes for 17 brain regions, which include grey matter from the cortex, deep grey matter and normal appearing white matter, as listed in Supplementary Table 1; and (iii) the T1-weighted/T2-weighted ratio on the same target regions after removing lesion voxels as a proxy for normal-appearing microstructural tissue damage.^28^ We used lesion volume over T2 lesion or gadolinium-enhancing lesion counts as continuous variables for modelling purposes. The full list of the resulting 35 MRI-extracted variables is detailed in Supplementary Table 2. We visually assessed the quality of all the MRI processing outputs (i.e. lesion masks, neuroanatomical segmentation and T1/T2 ratio maps) for outliers and erroneous segmentations. We will explain these steps in more detail.

#### T2-hyperintense lesion volume assessment

We corrected all scans for field inhomogeneity (bias field correction)^29^ and reduced noise using a spatially adaptive filter.^30^ We aligned the T1-weighted image to the Montreal Neurological Institute (MNI) template,^31^ and extracted a brain mask using the ROBEX tool,^32^ which is then used as input for the Atropos algorithm to segment the white matter.^33^ We aligned the T2-FLAIR image from the same MRI session to the T1-weighted image, and we normalized the intensities of both images using the white matter mask with the Fuzzy C-Means method.^34^ Next, we generated T2-hyperintense lesion masks using the DeepMedic convolutional neural network^35^ on the normalized images. This process was applied only to the training set, because the external test dataset already did not have T2-FLAIR (instead, we used manually annotated T2-hyperintense lesion masks).^23^ Finally, we calculated the total lesion volume (in millimetres cubed) from these masks.

#### Brain volumetric analysis

We segmented each bias-corrected T1-weighted MRI using a multi-atlas approach. Initially, we used the lesion masks to fill hypointense areas in the T1-weighted scan. We applied non-linear registration^36^ to align each T1-weighted MRI with 50 labelled templates from the MindBoggle atlas.^36^ We then projected these labels onto the original T1-weighted space using the inverse transform. We used a joint label fusion method^37^ to generate consensus labels. The resulting segmentation allowed us to locate and measure volume (in millimetres cubed) of each target region.

#### T1/T2 ratio

To calculate the T1-weighted/T2-weighted ratio, we first co-registered each T2-weighted image to its corresponding T1-weighted scan from the same MRI session using affine transformation.^31^ We then calibrated the intensities of both images through a linear scaling procedure (see the Supplementary material) to standardize their scale. The voxel-wise ratio of these calibrated images yielded the T1-weighted/T2-weighted ratio image. We identified each target brain region within the ratio image using the segmentation derived from the T1-weighted MRI (as described in the ‘Brain volumetric analysis’ section). We computed the median T1-weighted/T2-weighted value for each region.

#### Percentage brain volume change and gadolinium-enhancing lesions

We used percentage brain volume change (PBVC) and gadolinium-enhancing lesions from previously published clinical trial results.^22,23^

### Model development

#### The MRI–sNfL model

##### Feature selection

To select an optimal subset of MRI-derived variables for the new SuStaIn model, we implemented a two-stage selection process. Initially, from a pool of 35 available variables (Supplementary Table 2), we identified the 10 variables that exhibited the strongest correlation with EDSS in the training set, with the correlation matrix presented in Supplementary Fig. 1. Subsequently, recursive feature elimination^38^ was applied to this 10-variable subset to distil the selection further, to five variables. The objective of the recursive feature elimination procedure was to identify the feature combination that optimized the correlation between the stages of the resulting SuStaIn models and EDSS. The specific number of variables retained at each stage, 10 and five, respectively, was an arbitrary decision guided by several considerations: the size of the available training dataset (*n* = 189), the model parsimony and interpretability, and the high collinearity among many MRI-derived metrics.

##### Feature preprocessing

To summarize, we normalized anatomical volumes, T1-weighted/T2-weighted and lesion variables into *z*-scores. For volumes and T1/T2, we used the Human Connectome Project^39^ dataset as the reference distribution. The Supplementary material describes the standardization process for the lesion volume. The sNfL measurements were already laboratory standardized and provided as age-adjusted *z*-scores against a control population, as explained elsewhere.^27^

##### Model training

We applied the SuStaIn algorithm to the first visit at the study entry for each participant in the training set. We configured the algorithm to discover models with one, two or three data-derived subtypes. We conducted a 5-fold cross-validation to select the optimal number of subtypes by calculating, for each configuration, log-likelihood and cross-validation information criteria (CVIC), as defined by Young *et al*.^7^ Based on these metrics, we selected the optimal model and used it to assign a data-derived subtype and stage to each participant at every visit across both training and external test datasets. We refer to this combined MRI and sNfL model as the MRI–sNfL model.

#### MRI-only model

As the primary benchmark for this study, and to assess whether incorporating sNfL levels improves the prediction of clinically meaningful outcomes in comparison to extensively characterized and validated MRI-derived MS subtypes, we used the previously published SuStaIn MRI-only model,^8^ which is publicly available.^40^ This model incorporated 13 MRI-derived variables: volumetric measurements of deep grey matter, frontal, occipital, parietal, temporal lobes and limbic cortex; total lesion volume; and T1/T2 ratios from specific white matter areas (cerebellar, temporal, cingulate, corpus callosum and temporal–parietal). We will refer to this existing model as the MRI-only model. This model classified participants into a lesion-dominant pattern (lesion-led), those with early abnormality in normal-appearing white matter T1/T2 ratio (NAWM-led) and those with early atrophy in the grey matter (GM-led).^8^

#### 5-MRI model

To evaluate the added value of sNfL better, we trained a SuStaIn model using only the five MRI variables included in the MRI–sNfL model. We refer to this reduced-variable model as the 5-MRI model. The same feature preprocessing pipeline as used in the MRI–sNfL model was applied. The algorithm was configured to identify up to two subtypes, consistent with the MRI–sNfL model, which we generically refer to as ‘MRI Subtype 1’ and ‘MRI Subtype 2.’ The 5-MRI model was then used to classify and stage each participant in both the training and external test sets.

### Model testing

#### MRI feature harmonization

It has been shown that quantitative measures directly dependent on the MRI intensity profile, such as the segmented structures and T1-weighted/T2-weighted ratio, are sensitive to inter-study differences (e.g. different MRI scanners or acquisition parameters).^41^ To mitigate the potential confounder, we used the ComBat algorithm^42^ to harmonize the volumes and T1-weighted/T2-weighted variables from the external test dataset with respect to the training data.

#### Associations with longitudinal MRI and clinical outcomes

We analysed outcomes across data-derived subtypes using longitudinal visits from training and external testing datasets. Using linear mixed-effects models with random-effects intercepts, we estimated average change rates for three outcomes (EDSS, sNfL and gadolinium-enhancing lesion counts) annually, with subject identity as the random effect. We analysed these in two ways. First, we fitted separate mixed-effects models for each SuStaIn subtype group. Second, we fitted separate models for each SuStaIn subtype–treatment interaction term to assess potential treatment effects. We used the Statsmodel Python package (version 0.13.5).

#### Correlation at screening (study entry) between SuStaIn stage and clinical outcomes

We assessed how SuStaIn stage relates to three variables (chronological age, EDSS and the number of active lesions) using Spearman’s rank correlation coefficient (α = 0.05). We performed the analysis separately for the MRI-only and the MRI–sNfL models. For each correlation, we computed the coefficient and its two-tailed *P*-value with SciPy (v.1.10.0). To compare two independent correlation coefficients, we applied Fisher’s *z*-test. Based on Schober *et al*.,^43^ we classified correlations as weak (ρ = 0.10–0.39), moderate (ρ = 0.40–0.69), strong (ρ = 0.70–0.89) or very strong (ρ = 0.90–1.00).

#### Analysis of percentage brain volume change

We use the PBVC between the baseline and final follow-up MRI scans (Month 6 in the training set, and Month 24 (control) or Month 60 (treatment) in the external test set) and express it as an annualized rate of change for analysis.

#### Survival analysis on the risk of developing new gadolinium-enhancing lesions

We used a Cox proportional hazards model to assess the risk of developing a new gadolinium-enhancing lesion, with predictors SuStaIn subtype (MRI–sNfL model) and treatment assignment. An increase in lesion count at any follow-up visit was treated as an event at that visit. We estimated hazard ratios and 95% confidence intervals (CI) with Lifelines (v.0.27.8). We did not analyse time to EDSS worsening because the event rate was too low (training set 5.6%; external test set 43.6%).

## Results

### Participant characteristics

In the training set, 161 participants (85%) had relapsing–remitting MS and 28 (15%) had secondary progressive MS; 69% were women (Table 1). The mean age of the cohort was 42 ± 10 years, the mean time since diagnosis 7.7 ± 6.4 years and the median screening EDSS 3. In contrast, the external test set comprised individuals evaluated at the point of diagnosis of MS or clinically isolated syndrome. Among them, 284 (64%) were women, the mean age was 31 ± 8 years and the median EDSS 1.5. Baseline brain volume averaged 1177 ± 123 ml in the training set and 1158 ± 113 ml in the external test set.

**Table 1:** Demographics and screening characteristics of participants

Characteristic	Training	External test
Number of subjects	189	445
Number of visits	799	1214
MS phenotype	RRMS (*n* = 161, 85%) SPMS (*n* = 28, 15%)	Newly diagnosed relapsing–remitting MS and clinically isolated syndrome (*n* = 445, 100%)
Self-reported sex	Female (*n* = 131, 69%) Male (*n* = 58, 31%)	Female (*n* = 284, 64%) Male (*n* = 161, 36%)
Median age, years (interquartile range)	41 (34, 50)	30 (24, 37)
Median disease duration, years from diagnosis (interquartile range)	6.5 (2.3, 12.1)	Newly diagnosed
Prior treatments	77.2% no prior treatment 17.5% moderate-efficacy treatment 5.3% high-efficacy treatment	No prior treatment
Treatment arms	Placebo (*n* = 50) Evobrutinib 25 mg QD (*n* = 46) 75 mg QD (*N* = 47) 75 mg BID (*n* = 46)	Placebo (*n* = 144) Interferon-beta 1a 44 μg OW (*n* = 152) Interferon-beta 1a 44 μg TIW (*n* = 149)
Median number of relapses within 2 years of study commencement	2 (1, 2)	No prior relapses
Median screening sNfL *z*-score (interquartile range)	1.28 (−0.12, 2.20)	2.01 (0.54, 3.13)
Median EDSS (interquartile range)	3.0 (2.0, 4.5)	1.5 (1.0, 2.0)
Median number of gadolinium-enhancing lesions (interquartile range)	0 (0, 1)	4 (1, 9)

Classification of prior treatments into moderate and high efficacy is detailed in Supplementary Table 5. Abbreviations: BID = bis in die (twice daily); MS = multiple sclerosis; OW = once weekly; QD = quaque die (once daily); RRMS = relapsing-remitting multiple sclerosis; sNfL = serum neurofilament light chain; SPMS = secondary progressive multiple sclerosis; TIW = three times weekly.

### MRI–sNfL model results

#### Selected variables

This model used five MRI variables: the limbic cortex, deep grey matter, parietal cortex, total lesion volumes and corpus callosum white matter T1-weighted/T2-weighted. Combined with the serum biomarker sNfL, the MRI–sNfL model had six variables.

#### The chosen model discovered subtypes characterized by early-sNfL and late-sNfL activity

We compared models with one, two or three data-derived subtypes. The two-subtype model (CVIC = 3195, log-likelihood = −318.7) and the three-subtype model (CVIC = 3189, log-likelihood = −318.22) fitted the data better than the single-subtype model (CVIC = 3316, log-likelihood = −330.8). We favoured the two-subtype model owing to its parsimony over the three-subtype model. It offered a simpler explanation of the data while still effectively differentiating between data-derived subtypes, whereas two subtypes in the three-subtype model had highly similar patterns in their sequence (stage), as shown in Supplementary Fig. 5. Given that each variable can progress through three stages (mild, intermediate and severe) based on where each variable was placed on the reference distribution (1, 2 or 3 standard deviations from the mean), the eventual model staged patients into 18 stages (six variables multiplied by three levels of abnormality).

**Figure 2:**
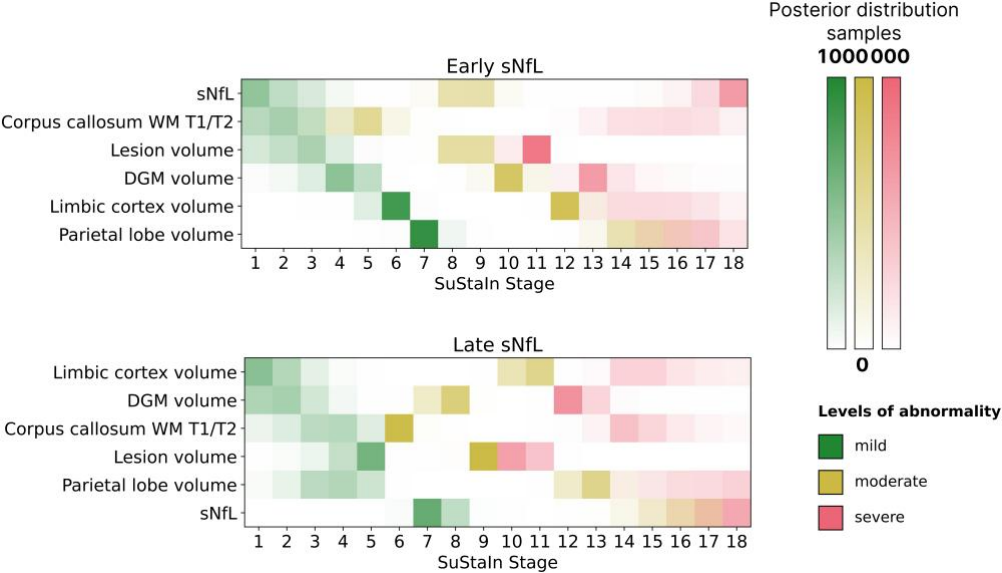
**Order of progression of abnormality in the early-sNfL and late-sNfL subtypes.** Positional variance plot of early-sNfL and late-sNfL subtypes shows the level of uncertainty in assigning each biomarker listed in the vertical axis with a certain stage shown on the horizontal axis. The three colours show mild, moderate and severe abnormalities (*z*-scores of 4, 6 and 8). The colour shades for each level of abnormality show the associated uncertainty in the model. The early-sNfL subtype is characterized by elevated sNfL levels, reduction of normal-appearing corpus callosal T1/T2 ratio, and lesion accrual as early events among the variables examined. In contrast, the late-sNfL subtype showed early volume loss in the limbic cortex and deep grey matter, with sNfL elevation occurring at later stages of multiple sclerosis worsening. Feature order is based on the progression sequence in the early-sNfL subtype to aid interpretability. DGM = deep grey matter; sNfL = serum neurofilament light (chain); SuStaIn = subtype and stage inference; WM = white matter.

Figure 2 shows the positional variance plot of two subtypes, which we termed early-sNfL and late-sNfL subtypes. The early-sNfL subtype is characterized by elevated sNfL levels, reduction of normal-appearing corpus callosal T1/T2 ratio and lesion accrual as early events among the variables examined. In contrast, the late-sNfL subtype showed early volume loss in the limbic cortex and deep grey matter, with sNfL elevation occurring at later stages of abnormality accumulation. The demographic and clinical characteristics of both subtypes are provided in Table 2. For both the training and external test datasets, subtypes exhibit age differences (*P* < 0.001). Specifically, late sNfL was more prevalent among older subjects, and in the training set, was more disabled. Supplementary Fig. 4 illustrates the proportions of subtypes stratified by prior treatment efficacy, for which we found no significant association with subtype assignment (χ^2^ test, *P* = 0.24).

**Table 2:** Demographic and clinical data by subtype from the MRI–sNfL model

Parameter	Training			External test		
	Early-sNfL	Late-sNfL	*t*-test	Early-sNfL	Late-sNfL	*t*-test
Sample, *n* (%)	74 (44%)	93 (56%)	–	267 (61%)	168 (39%)	–
Females in each subtype, *n* (%)	36 (49%)	77 (83%)	–	(155) 58%	123 (73%)	–
Average age, years (mean ± standard deviation)	37 ± 9	46 ± 9	*P* < 0.001	30 ± 8	34 ± 8	*P* < 0.001
MS types, *n* (%)	RRMS = 63 (85%) SPMS = 11 (15%)	RRMS = 78 (84%) SPMS = 15 (16%)	–	RRMS = 267 (100%)	RRMS = 168 (100%)	–
Median EDSS, median (IQR)	2.5 (1.5, 3.5)	3.5 (2.0, 4.5)	*P* < 0.001	1.5 (1, 2)	1.5 (1, 2)	*P* = 0.083

Clinical and demographic characteristics are presented for subtypes derived from the MRI–sNfL model (early-sNfL, late-sNfL). Data are shown separately for the training and external test datasets.Abbreviations: EDSS = expanded disability status scale; IQR = interquartile range; MS = multiple sclerosis; RRMS = relapsing-remitting multiple sclerosis; sNfL = serum neurofilament light chain; SPMS = secondary progressive multiple sclerosis.

At the screening visit in the training dataset, 93 participants (56%) were assigned to the late-sNfL subtype and 74 (44%) to the early-sNfL subtype. During follow-up, 48 individuals (∼29%) changed subtypes. Among those with high-confidence assignments (*n* = 95, probability >85%), only seven participants (∼7%) switched subtypes. In the external test dataset, 267 participants (61%) were initially classified as early-sNfL and 168 (39%) as late-sNfL. Over time, 177 individuals (41%) changed subtypes, reduced to 66 (23%) among those with high-confidence assignments (*n* = 291). To assess the sensitivity of this stability to the confidence threshold, Supplementary Fig. 3 shows a sensitivity analysis using thresholds of 80%, 85% and 90%. The results highlight that subtype switching consistently decreases as the threshold increases, indicating that higher-confidence assignments are more stable.

### Subtypes from the MRI-only model

In the training set, our previously published MRI-only model classified 119 patients (56%) as lesion-led, 60 (28%) as grey matter (GM)-led, and the remainder were normal-appearing white matter- or NAWM-led. In the external test dataset, 221 (50%) were lesion-led, 152 (34%) GM-led and the rest NAWM-led. Supplementary Table 3 shows the demographic characteristics of subtypes classified by this model in both cohorts.

### Subtypes from the 5-MRI model

Supplementary Fig. 2 depicts the progression patterns of the two identified MRI subtypes. Both Subtype 1 and Subtype 2 show an initial decline in the T1/T2 ratio of normal-appearing corpus callosal white matter. Subtype 1 is characterized by early deep grey matter volume loss, whereas Subtype 2 exhibits early lesion accumulation. Supplementary Table 4 presents the demographic characteristics of participants assigned to each subtype in both cohorts.

### Subtypes and stages correlated with demographic and disability variables

We refer to the stages derived from the MRI–sNfL model as ‘MRI–sNfL model stages’, those from the MRI-only model as ‘MRI-only model stages’ and those from the 5-MRI model as ‘5-MRI model stages’. All correlation coefficients between the SuStaIn stages derived from each of these models and clinical measures at study entry are summarized in Table 3.

**Table 3:** Demographic and clinical data and their correlations with data-derived stages across the different models

Parameter	Training			External test		
	Spearman correlation with MRI–sNfL model stages					
	All	Early-sNfL	Late-sNfL	All	Early-sNfL	Late-sNfL
Age	0.347 (*P* < 0.001)	0.361 (*P* = 0.002)	0.343 (*P* < 0.001)	0.031 (*P* = 0.517)	0.125 (*P* = 0.041)	0.126 (*P* = 0.102)
EDSS	0.420 (*P* < 0.001)	0.396 (*P* < 0.001)	0.457 (*P* < 0.001)	0.163 (*P* < 0.001)	0.196 (*P* = 0.001)	0.075 (*P* = 0.336)
Number of active lesions	0.093 (*P* = 0.234)	0.115 (*P* = 0.330)	0.109 (*P* = 0.301)	0.636 (*P* < 0.001)	0.571 (*P* < 0.001)	0.564 (*P* < 0.001)

	Spearman correlations with MRI-only model stages							
	All	Lesion-led	GM-led	NAWM-led	All	Lesion-led	GM-led	NAWM-led
Age	0.275 (*P* < 0.001)	0.230 (*P* = 0.018)	−0.025 (*P* = 0.858)	0.276 (*P* = 0.132)	0.141 (*P* = 0.003)	0.228 (*P* < 0.001)	−0.026 (*P* = 0.753)	0.106 (*P* = 0.375)
EDSS	0.231 (*P* = 0.001)	0.278 (*P* = 0.004)	−0.272 (*P* = 0.049)	0.501 (*P* = 0.004)	0.067 (*P* = 0.159)	0.112 (*P* = 0.099)	0.029 (*P* = 0.727)	0.172 (*P* = 0.149)
Number of active lesions	−0.020 (*P* = 0.785)	−0.063 (*P* = 0.524)	0.004 (*P* = 0.977)	0.202 (*P* = 0.276)	0.223 (*P* < 0.001)	0.367 (*P* < 0.001)	0.174 (*P* = 0.033)	0.355 (*P* = 0.002)

	Spearman correlation with 5-MRI model stages					
	All	MRI subtype 1	MRI subtype 2	All	MRI subtype 1	MRI subtype 2
Age	0.402 (*P*< 0.001)	0.326 (*P* < 0.001)	0.603 (*P* < 0.001)	0.185 (*P* < 0.001)	0.122 (*P* = 0.037)	0.329 (*P* < 0.001)
EDSS	0.419 (*P* < 0.001)	0.428 (*P* < 0.001)	0.431 (*P* = 0.002)	0.114 (*P* = 0.017)	0.094 (*P* = 0.108)	0.173 (*P* = 0.036)
Number of active lesions	0.027 (*P* = 0.710)	0.044 (*P* = 0.606)	0.055 (*P* = 0.715)	0.487 (*P* < 0.001)	0.553 (*P* < 0.001)	0.427 (*P* < 0.001)

Spearman correlation coefficients between model-inferred disease stages and clinical variables (age, EDSS and number of active lesions) are reported for each subtype across the MRI–sNfL, MRI-only and 5-MRI models. Analyses are separated by training and external test datasets. Subtypes include early-sNfL and late-sNfL (MRI–sNfL model), Lesion-led, GM-led and NAWM-led (MRI-only model), and Subtypes 1 and 2 (5-MRI model).Abbreviations: EDSS = expanded disability status scale; GM = grey matter; NAWM = normal appearing white matter; sNfL = serum neurofilament light chain.

Regarding EDSS, the MRI–sNfL model stages in the training set showed a stronger correlation (ρ = 0.420, *P* < 0.001) compared with the MRI-only model (ρ = 0.231, *P* = 0.001). In the external test dataset, the MRI–sNfL model maintained a weak but significant correlation (ρ = 0.163, *P* < 0.001), whereas the MRI-only model showed no significant correlation (ρ = 0.067, *P* = 0.159). The MRI–sNfL subtypes in the training set both showed moderate correlations with EDSS: late-sNfL (ρ = 0.457, *P* < 0.001) and early-sNfL (ρ = 0.396, *P* < 0.001). In the external test set, only early-sNfL maintained a statistically significant correlation (ρ = 0.196, *P* = 0.001), whereas late-sNfL did not (ρ = 0.075, *P* = 0.336). The 5-MRI model stage was correlated with EDSS in the training set (ρ = 0.419, *P* < 0.001), similar in strength to the MRI–sNfL model, but in the external test set the correlation was weaker (ρ = 0.114, *P* = 0.017), suggesting that the MRI–sNfL model generalized better.

For chronological age, both MRI–sNfL (ρ = 0.347, *P* < 0.001) and MRI-only (ρ = 0.275, *P* < 0.001) stages showed weak correlations in the training set. In the external test set, the MRI–sNfL model showed no significant overall correlation (ρ = 0.031, *P* = 0.517), although early-sNfL showed a weak but significant correlation (ρ = 0.125, *P* = 0.041). The MRI-only model, in contrast, retained a weak but significant correlation in the external test set (ρ = 0.141, *P* = 0.003). The 5-MRI model showed stronger correlations with age in comparison to the other models in both datasets: in the training set (ρ = 0.402, *P* < 0.001) and external test set (ρ = 0.185, *P* < 0.001).

Regarding active lesions, the MRI–sNfL model showed no significant correlation in the training set (ρ = 0.093, *P* = 0.234), but demonstrated a moderate correlation in the external test set (ρ = 0.636, *P* < 0.001), with both early-sNfL (ρ = 0.571, *P* < 0.001) and late-sNfL (ρ = 0.564, *P* < 0.001) subtypes contributing similarly. The MRI-only model showed no correlation in the training set, but a weak significant correlation in the external test set (ρ = 0.223, *P* < 0.001). The 5-MRI model showed no correlation with active lesions in the training set, but a moderate correlation in the external test set (ρ = 0.487, *P* < 0.001), although this was weaker than that of the MRI–sNfL model.

### Longitudinal analysis of gadolinium-enhancing lesion counts in SuStaIn subtypes

Table 4 reports results from linear mixed-effects models estimating annual changes in EDSS, lesion counts and sNfL levels by subtype and treatment group. In the treatment group of the training set, subjects classified as early-sNfL showed a significantly faster reduction in active lesion counts (β = −4.9568, 95% CI −8.741, −1.173, *P* = 0.01) compared with the late-sNfL subtype (β = 1.1053, 95% CI −2.039, −0.172, *P* = 0.02). We found no significant differences in the control group of the training set.

**Table 4:** Longitudinal analysis for each subtype

Variable	Subtype	Treatment group	Training	*P*-value	External Test	*P*-value
EDSS	Early-sNfL	Both	0.190 [0.014, 0.367]	**0**.**035**	−0.031 [−0.051, −0.01]	**0**.**004**
		Control	0.277 [−0.034, 0.587]	0.080	−0.241 [−0.328, −0.154]	**<0**.**001**
		Treatment	0.086 [−0.041, 0.213]	0.183	−0.021 [−0.042, 0.001]	0.057
	Late-sNfL	Both	−0.107 [−0.195, −0.019]	**0**.**017**	−0.017 [−0.043, 0.01]	**0**.**222**
		Control	−0.176 [−0.311, −0.040]	**0**.**011**	−0.246 [−0.36, −0.132]	**<0**.**001**
		Treatment	−0.028 [−0.134, 0.078]	0.603	−0.003 [−0.03, 0.024]	0.840
Active lesion count	Early-sNfL	Both	−2.002 [−4.527, 0.524]	0.120	0.223 [0.065, 0.382]	**0**.**006**
		Control	0.628 [−2.694, 3.950]	0.711	0.513 [−0.109, 1.135]	0.106
		Treatment	−4.957 [−8.741, −1.173]	**0**.**010**	0.212 [0.047, 0.378]	**0**.**012**
	Late-sNfL	Both	−0.515 [−1.046, 0.015]	0.057	−0.046 [−0.134, 0.042]	0.308
		Control	0.055 [−0.484, 0.593]	0.842	0.393 [0.142, 0.644]	**0**.**002**
		Treatment	−1.105 [−2.039, −0.172]	**0**.**020**	−0.079 [−0.175, 0.017]	0.106
sNfL	Early-sNfL	Both	−0.909 [−1.282, −0.536]	**<0**.**001**	−1.071 [−1.173, −0.968]	**<0**.**001**
		Control	−0.439 [−0.806, −0.072]	**0**.**019**	−0.929 [−1.109, −0.748]	**<0**.**001**
		Treatment	−1.489 [−2.157, −0.822]	**<0**.**001**	−1.131 [−1.256, −1.006]	**<0**.**001**
	Late-sNfL	Both	−0.128 [−0.517, 0.261]	0.518	−0.156 [−0.262, −0.050]	**0**.**004**
		Control	−0.087 [−0.680, 0.506]	0.774	−0.108 [−0.300, 0.084]	0.27
		Treatment	−0.176 [−0.663, 0.311]	0.479	−0.180 [−0.307, −0.053]	**0**.**005**
PBVC, %	Early-sNfL	Both	−1.612 [−2.543, −0.680]	0.8	−0.463 [−0.529, −0.396]	**0**.**002**
	Late-sNfL		−1.465 [−2.205, −0.725]	–	−0.311 [−0.373, −0.249]	–
	Early-sNfL	Control	−0.976 [−2.235, 0.283]	0.47	−0.544 [−0.686, −0.403]	**0**.**015**
	Late-sNfL		−1.552 [−2.560, −0.545]	–	−0.305 [−0.424, −0.186]	–
	Early-sNfL	Treatment	−2.225 [−3.634, −0.816]	0.33	−0.407 [−0.463, −0.350]	**0**.**038**
	Late-sNfL		−1.363 [−2.507, −0.220]	–	−0.315 [−0.381, −0.250]	–

Longitudinal analysis for clinical variables Expanded Disability Status Scale (EDSS), number of active lesions, serum neurofilament light chain (sNfL) and percentage brain volume change (PBVC). PBVC was measured between screening MRI and follow-up MRI (Month 6 for the training set, Month 36 for the external test dataset). This was also further divided into control and treatment subgroups for both training and external test datasets. We analysed EDSS, active lesion count, and sNfL using mixed-effects models. Yearly trends, confidence intervals, and *P*-values are provided for these variables. PBVC is reported as an annual rate along confidence intervals. For PBVC, the *P*-values result from *t*-tests assessing statistically significant differences among subtypes distributions. Similar analysis for the MRI-only model is available in Supplementary Table 6.

In the treatment group of the external test dataset, the late-sNfL subtype showed no significant change in gadolinium-enhancing lesion count (β = −0.079, 95% CI −0.175, 0.017, *P* = 0.106), whereas the early-sNfL subtype had a significant increase (β = 0.212, 95% CI 0.047, 0.378, *P* = 0.012). In the control group, both the early-sNfL (β = 0.513, 95% CI −0.109, 1.135, *P* = 0.106) and late-sNfL (β = 0.393, 95% CI 0.142, 0.644, *P* = 0.002) subtypes showed positive rates of active lesion accrual, although statistical significance was not reached for the early-sNfL subtype.

### Longitudinal analysis of sNfL in data-derived subtypes

Analysis of annual sNfL trends in the training set showed significant reductions in the early-sNfL subtype for both control (β = −0.4388, 95% CI −0.806, −0.072, *P* = 0.019) and treatment (β = −1.4894, 95% CI −2.157, −0.822, *P* < 0.001) groups, whereas the late-sNfL subtype showed no significant changes. The external test dataset confirmed these patterns: early-sNfL subjects showed reduced levels over trial years in both control (β = −0.9286, 95% CI −1.109, −0.748, *P* < 0.001) and treatment (β = −1.1311, 95% CI −1.256, −1.006, *P* < 0.001) groups. Additionally, treated late-sNfL subjects in the external test dataset demonstrated a modest but significant decrease in sNfL (β = −0.1798, 95% CI −0.307, −0.053, *P* = 0.005).

### Comparison of atrophy rates between subtypes

In the training set, as shown in Table 4, PBVC values were similar between early-sNfL (−1.612% ± 3.4460%) and late-sNfL (−1.465% ± 3.3266%, *P* = 0.805) groups. In the external test dataset, the early-sNfL subtype had a faster rate of brain volume loss than the late-sNfL group (−0.463% ± 0.4263% versus −0.305% ± 0.4157%, *P* = 0.002).

As Table 4 shows, in the training set, we found no statistically significant differences in PBVC values between early-sNfL and late-sNfL groups in both control (−0.976% ± 3.182% versus −1.552% ± 3.274%, *P* = 0.471) and treatment (−2.225% ± 3.634% versus −1.363% ± 3.429%, *P* = 0.332) groups.

In the external test dataset, early-sNfL participants showed a statistically significant faster rate of PBVC than the late-sNfL subtype in the treatment group (−0.407% ± 0.2773% versus −0.315% ± 0.2675%, *P* = 0.038) and in the control group (−0.544% ± 0.5720% versus −0.305% ± 0.4157%, *P* = 0.015).

### Time to radiological disease activity

**Figure 3:**
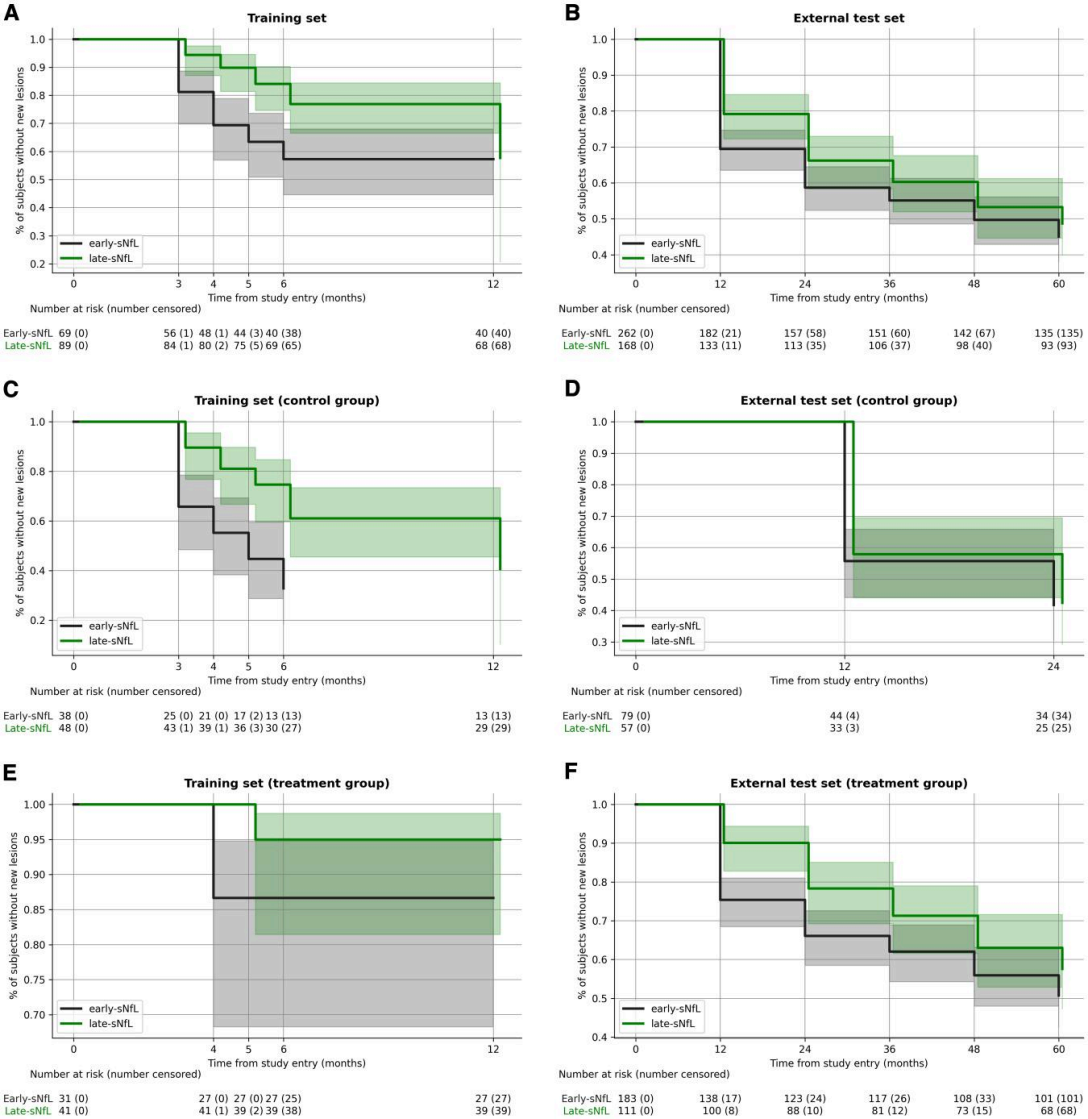
**Kaplan–Meier plots showing the time to new lesions in the training and external test dataset, stratified by subtype and treatment group.** (**A** and **B**) Early-sNfL and late-sNfL subtypes in the training (**A**) and external test (**B**) datasets. (**C** and **D**) Control group: early-sNfL and late-sNfL subtypes in training (**C**) and external test (**D**) datasets. Note that in **C**, the early-sNfL curve ends at Month 6 because there were no follow-up data available at Month 12 for the remaining subjects in this group. (**E** and **F**) Treatment group: early-sNfL and late-sNfL subtypes in the training (**E**) and external test (**F**) datasets. The early-sNfL subtype exhibits an increased risk of developing new lesions across both datasets relative to the late-sNfL subtype. The treated subtypes show a reduced risk of new lesions in comparison to the control groups in both datasets. The expected time to event was longer in the late-sNfL subtype than the early-sNfL subtype in both training and external test datasets, although this is most clearly shown in the training dataset. sNfL = serum neurofilament light (chain).

Figure 3 presents Kaplan–Meier plots for the training and external test datasets, stratified by MRI–sNfL subtype with and without treatment groups. After adjusting for treatment effects, we found that the early-sNfL group had a 144% increase in the risk of new lesion development in the training set compared with the late-sNfL group (hazard ratio = 2.44, 95% CI 1.38, 4.30, *P* < 0.005). Similar trends were observed in the external test dataset, where early-sNfL classification had, on average, higher risk of lesion development by 22% (hazard ratio = 1.22, 95% CI 0.92, 1.63), although this finding did not reach statistical significance (*P* = 0.17). Treatment effects were observed across both MRI–sNfL subtypes and in both the training and external test datasets. These results are summarized in Table 5.

**Table 5:** Risk of developing new lesions in data-derived subtypes

	Training set	External test set
**With treatment group adjustment, hazard ratio [95% confidence intervals] (*P*-value)**		
Early-sNfL versus late-sNfL	2.44 [1.38, 4.30] (*P* < 0.005)	1.22 [0.92, 1.63] (*P* = 0.17)
**Treated versus controls**		
Early-sNfL	0.13 [0.05, 0.38] (*P* < 0.001)	0.48 [0.33, 0.71] (*P* < 0.001)
Late-sNfL	0.10 [0.02, 0.44] (*P* = 0.002)	0.27 [0.16, 0.46] (*P* < 0.001)

Cox regression analysis results within subtypes derived in this study, termed early-sNfL and late-sNfL, show the risk of developing new gadolinium-enhancing lesions based on assigned treatment groups was consistently higher in early-sNfL group. Hazard ratios are reported with 95% confidence intervals and *P*-values. Similar analysis for the MRI-only model is available in Supplementary Table 7.Abbreviation: sNfL = serum neurofilament light chain.

## Discussion

This study identified two data-derived MS subtypes, distinguished by the timing of sNfL elevation within an evolving landscape of MRI-derived abnormalities. Termed ‘early-sNfL’ and ‘late-sNfL’ subtypes, these categories reflect distinct biological profiles: the early-sNfL subtype displayed elevated sNfL levels, compromised corpus callosal integrity and lesion accrual early in the disease, consistent with active inflammatory and neurodegenerative processes manifesting in parallel. In contrast, the late-sNfL subtype began with tissue-specific volumetric loss (notably in the limbic cortex and deep grey matter) before sNfL levels became abnormal, suggesting a more insidious trajectory of neurodegeneration that precedes overt neuroaxonal injury. By integrating MRI and sNfL measures in a single unsupervised model, we have defined biologically grounded MS types that capture diverse disease pathways and their clinical implications.

We trained our model on a cohort that included both relapsing–remitting and secondary progressive MS (mean disease duration 7.7 years), then validated it in a larger, younger cohort of newly diagnosed MS (including clinically isolated syndrome), confirming generalizability at early stages. Our design identified subtypes spanning the entire disease course, essential for SuStaIn model training. As expected, the younger test cohort had more active disease by gadolinium-enhancing lesion count. Although these cohorts differed demographically and clinically (a typical source of bias in machine-learning models), SuStaIn differs from typical models not specifically designed for disease progression modelling and remains robust. SuStaIn assigns both a subtype and a stage to each patient, and therefore, our design and choice of cohorts with demographic differences allowed us to validate both the subtyping and staging by the model. Integration of sNfL with MRI improved correlations with disability (EDSS) and inflammation (enhancing lesions and sNfL), demonstrating that sNfL provides complementary information.

The newly identified MRI–sNfL subtypes demonstrated demographic and biological differences. The late-sNfL subtype included more women and older individuals, whereas the early-sNfL subtype comprised younger patients with more active disease. In comparison to the late-sNfL group, early-sNfL patients showed greater reductions in active lesion counts and sNfL levels over time, particularly among those receiving treatment. Although treatments such as interferons and evobrutinib have shown limited efficacy in clinical trials, the observed differences in treatment response across subtypes support the biological relevance of our subtyping approach. In both datasets, the early-sNfL subtype exhibited faster brain atrophy rates, probably reflecting more severe neurodegeneration driven by inflammation. Together, these findings suggest that the MRI–sNfL subtypes capture meaningful differences in disease activity, treatment response and progression risk.

Various pathophysiological biomarkers to classify MS disease course based on biological understanding have been proposed.^4-6,27,44,45^ Recent studies investigated MRI-based MS types with distinct patterns of brain volume loss correlated with clinical outcomes and sNfL independent of traditional clinical phenotypes, but have not used sNfL in subtyping tasks.^45^ Although MRI provides unique spatial information on the pathological spread, it lacks pathological specificity.^9^ Our new MRI–sNfL model represents a step change from our previous MRI-only approach, which offered valuable but incomplete insights into MS heterogeneity. MRI-based clustering revealed distinct subtypes driven by anatomical and microstructural features, but it could not fully capture the physiological processes driving disease activity and progression. By adding sNfL (an established indicator of neuroaxonal injury), we have advanced beyond the structural snapshot provided by MRI alone. Overall, our results support the added value of sNfL to MRI. When looking at the addition of sNfL to MRI in two MRI-only models (one previously published and one with the same five MRI variables), the MRI–sNfL model provided the highest correlation with active lesions, similar correlations with disability, but better generalization when comparing correlation coefficients in the training and external test set (the correlation coefficients declined for all models, but the absolute correlation coefficients remained highest for MRI–sNfL model). The decline in EDSS correlation between all models is attributable to the limited range of EDSS in the external test cohort of newly diagnosed MS and clinically isolated syndrome patients. In addition, stronger EDSS correlations in the training set compared with the testing set are observed as expected, owing to the use of EDSS-based feature selection within the training dataset.

sNfL has been linked to both active and chronic inflammation in earlier studies.^21,46-49^ The most consistent finding is its association with gadolinium-enhancing lesions, although radiological activity and sNfL rises do not always coincide.^21^ Srpova *et al*.^48^ also showed that, in some patients, sNfL elevation precedes brain atrophy. Our results corroborate the link between active lesions and high sNfL and newly show that elevated sNfL helps to stratify patients by inflammatory profile and the temporal evolution of sNfL and MRI abnormalities in subgroups of patients.

In training and external test datasets, the early-sNfL patients showed similar patterns compared with the late-sNfL (younger in early-sNfL and more women in the late sNfL) and, importantly, a similar proportion of patients with relapsing–remitting and secondary progressive MS in the training set with these two phenotypes. Our results underline that biology-grounded MS subtypes are largely independent of clinical course descriptors. These add to the evidence that biofluid biomarkers complement MRI to define the biological basis of MS disease evolution better.^12,14,27,50^ Longitudinal application of the model allows us to assess how subtype and stage assignments change over time. Although the model does not explicitly allow for deterministic transitions between subtypes, we occasionally observe ‘subtype switching’, whereby the most probable subtype assignment of a patient changes between visits. We interpret this as reflecting either uncertainty in subtype classification for borderline cases or genuine overlap between trajectories. Given that 7% of patients switched from one subtype to another in the training dataset, and 23% switched in the testing dataset, these subtypes are likely to represent a continuum of underlying pathology. SuStaIn captures this with probability-based membership, evidenced by the reduction in subtype switching when membership certainty was increased. Data-derived subtyping can, therefore, impact future disease course descriptions and prognosticate MS outcomes. From a clinical perspective, we can speculate that this might indicate a shift from a dominant inflammatory profile to a more neurodegenerative phase or vice versa. Future research with longer follow-ups can clarify this.

In the training cohort, patients classified in the early-sNfL subtype displayed a more rapid decline in gadolinium-enhancing lesions when treated, whereas those in the late-sNfL subtype did not show this rapid reduction. Interestingly, this subtype-specific effect was not seen in the control (untreated) group, suggesting that early-sNfL patients respond more robustly to treatment regarding reduction in active lesions. Conversely, in the test cohort, early-sNfL patients exhibited a faster decline in brain volume compared with late-sNfL patients, a finding not observed in the training data. Across both the training and test cohorts, early-sNfL patients also showed a higher likelihood of new lesion formation relative to late-sNfL patients. The inferred ‘stages’ of the MRI–sNfL model are a proxy for pathological accumulation that showed stronger associations with both EDSS and active lesions than our MRI-only model.

Within the treated cohorts, the early-sNfL subtype had a rapid gadolinium-enhancing lesion reduction, suggesting a more pronounced and immediate therapeutic effect in this subgroup. In contrast, the late-sNfL subtype did not have such a drop, indicating potential differences in the underlying disease mechanism. Meanwhile, in patients who were not on treatment, serum NfL levels still declined in both early- and late-sNfL subtypes over the course of the study. We attribute this reduction (despite the lack of active treatment) to the eligibility criteria of the clinical trials, which required participants to have ongoing disease activity (recent relapse or enhancing lesions). Consequently, this decrease might reflect ‘regression to the mean’ once the most active phase of inflammation subsides rather than a true therapeutic effect.^51^

Correlations between our model stages and EDSS were weak, mirroring the many reports of only weak biomarker–EDSS associations. This is expected, because EDSS is weighted towards motor function and captures only a narrow slice of MS burden, whereas MRI and serum biomarkers typically change before clinical symptoms become evident. Hence, attenuated cross-sectional correlations do not undermine the clinical promise of the model. Instead, they highlight the need for longer prospective studies that pair the model with broader outcome measures (cognition, quality of life and other patient-reported domains) beyond EDSS alone. Nonetheless, we can speculate that our model can provide staging of MS to facilitate future early interventions (before disability emerges) and simultaneously stratifies patients by their biomarker profiles, with potential to guide personalized therapy.

We should address several hurdles to bring our research model into everyday clinical care. Clinicians first need tools that convert routine MRI scans into precise measures of the brain structures of each patient. Although new tools are becoming available for brain MRI processing of real-world data,^52^ few hospitals have the infrastructure for these tasks. Even where such tools exist, the variability of everyday scans introduces new heterogeneity that demands further study. As sNfL is becoming widely available and digital infrastructure improves, we expect our multimodal model to evolve into future decision aid systems after further rigorous research on real-world data. Because subtype assignment is possible from a single cross-sectional scan, the model remains usable even when routine follow-up is sparse; our longitudinal analyses demonstrate that these baseline labels carry prognostic weight over time. Although harmonization can reduce variability across imaging sites, it also creates practical challenges for clinical adoption. Notably, the MRI–sNfL model retained strong performance even without harmonization (see Supplementary Table 8), supporting its use in settings with limited data or infrastructure.

Although the MRI–sNfL model provides an interpretable framework for subtyping and staging MS, it operates under several key assumptions. The SuStaIn algorithm models disease progression as a monotonic sequence of biomarker changes, with each subtype following a fixed order of abnormality accumulation. This assumption allows for tractable modelling of complex data but might limit sensitivity to fluctuating trajectories. To manage uncertainty, SuStaIn uses a Bayesian framework with Markov chain Monte Carlo sampling, which enables probabilistic estimates of both subtype and stage assignments. Although recent extensions of SuStaIn have introduced methods for accommodating missing data,^53^ all participants in our study had complete biomarker profiles, and missing data handling was not required here. These modelling assumptions should be considered when interpreting subtype assignments, particularly in broader clinical settings.

This study has several limitations. First, we drew our training and testing samples from clinical trial cohorts, which do not fully represent the broader MS population, including those with comorbidities or underrepresented ethnic groups and primary progressive MS, with strict eligibility criteria. As a result, for example, disability range was limited in the external testing dataset. Despite this, our MRI–sNfL model stages showed significant correlations with disability, showing the added value even with limited EDSS ranges. Therefore, future studies in diverse clinical settings are required for clinical translation. Second, our model was trained on both relapsing–remitting and secondary progressive cases, and is trained across the MS continuum, yet its accuracy in late-stage disease still warrants prospective validation, because our testing cohort was limited to early MS. Third, although adding sNfL enhanced the biological relevance of the model and, in some cases, clinical correlations, other fluid biomarkers or advanced imaging modalities (such as myelin-sensitive MRI sequences) will provide more comprehensive insights into disease progression. However, addition of more advanced measures reduces the accessibility and introduces obstacles to eventual clinical translation.

## Conclusion

Our findings advance the understanding of MS heterogeneity by revealing distinct biological trajectories rooted in MRI and fluid biomarkers. sNfL, despite being a non-specific marker related to neuronal cytoskeleton, complements MRI measures of disease activity and neurodegeneration. Integrating sNfL with MRI refines subtyping and provides a foundation for earlier, more individualized prognosis. Ultimately, this approach might pave the way for more targeted therapeutic strategies and improved patient outcomes.

## Supplementary Material

awaf331_Supplementary_Data

## Data Availability

The data supporting the findings of this study are proprietary and cannot be shared.
