# Supplementary material for: Combined magnetic resonance imaging and serum analysis reveals distinct multiple sclerosis types
Source: Brain. 2025 Dec 2;148(12):4578–91. doi: 10.1093/brain/awaf331 (PMC12678051; doi:10.1093/brain/awaf331)
Supplement: awaf331_Supplementary_Data [file awaf331_supplementary_data.pdf]

Supplementary material

Supplementary figures

Supplementary Figure 1. Correlation matrix of 13 MRI-derived variables, sNfL, and EDSS in the training set.

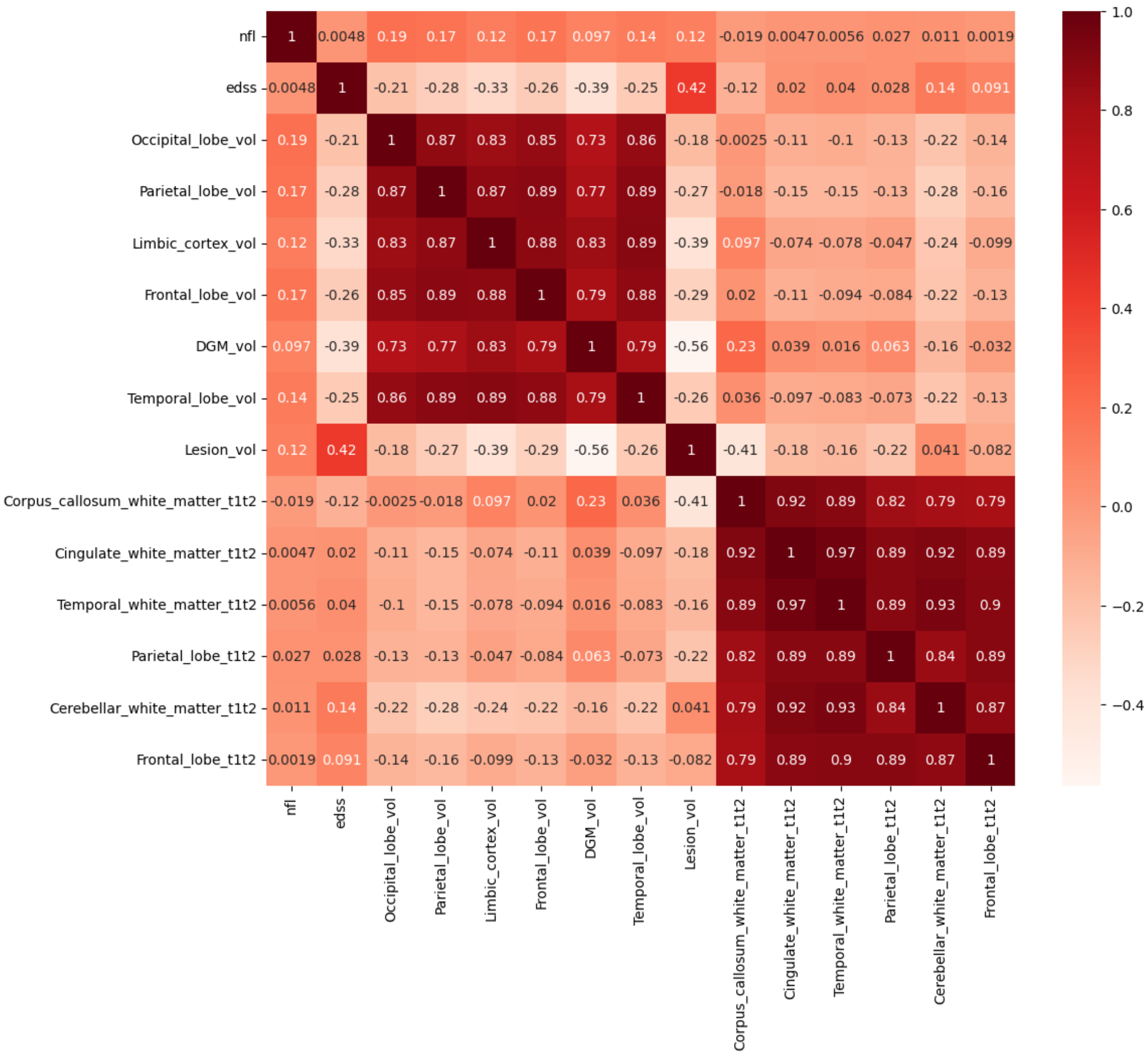

Caption: A correlation matrix showing the Spearman correlation coefficients in the training set among sNfL, the 13 MRI-derived variables used in the pre-trained model, and EDSS.

Supplementary Figure 2. Order of progression of abnormality in the two subtypes discovered from the SuStaIn model with 5 MRI variables.

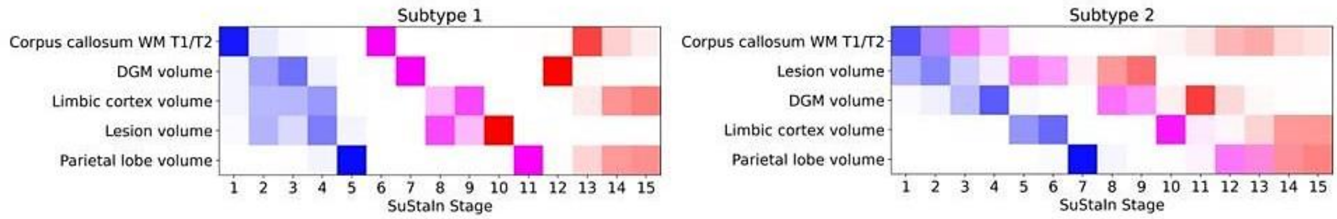

Caption: The positional variance plot, derived from five MRI variables and the SuStaIn model, illustrates the uncertainty in classifying each biomarker (listed vertically) to a specific disease stage (shown horizontally). Three colors represent mild, moderate, and severe abnormalities (corresponding to z-scores of 4, 6, and 8), with shading indicating the model's confidence in the classification at each stage. Both Subtype 1 and Subtype 2 exhibit an initial decline in the normal-appearing corpus callosal T1/T2 ratio. However, Subtype 1 is characterised by early deep grey matter volume loss, while Subtype 2 demonstrates early lesion accrual.

Abbreviations: WM, White Matter; DGM, Deep Grey Matter; SuStaIn, Subtype and Stage Inference;

Supplementary Figure 3. Sensitivity analysis of subtype assignment stability across varying confidence thresholds.

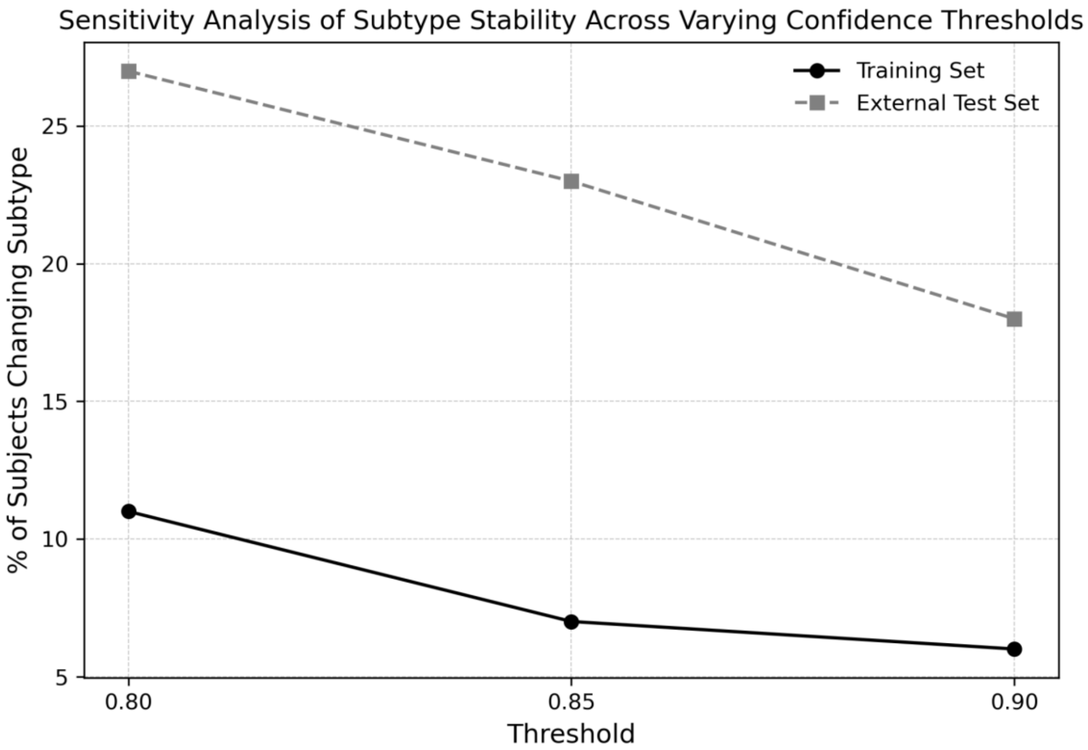

Caption: The plot shows the percentage of subjects whose subtype assignment changed over time at three different probability thresholds (80%, 85%, and 90%) for defining "high-confidence" assignments. As the confidence threshold increases, the proportion of subjects changing subtype decreases in both the training set (black line) and the external test set (grey dashed line).

Supplementary Figure 4: Distribution of MRI-sNfL subtypes by prior treatment efficacy in the training set.

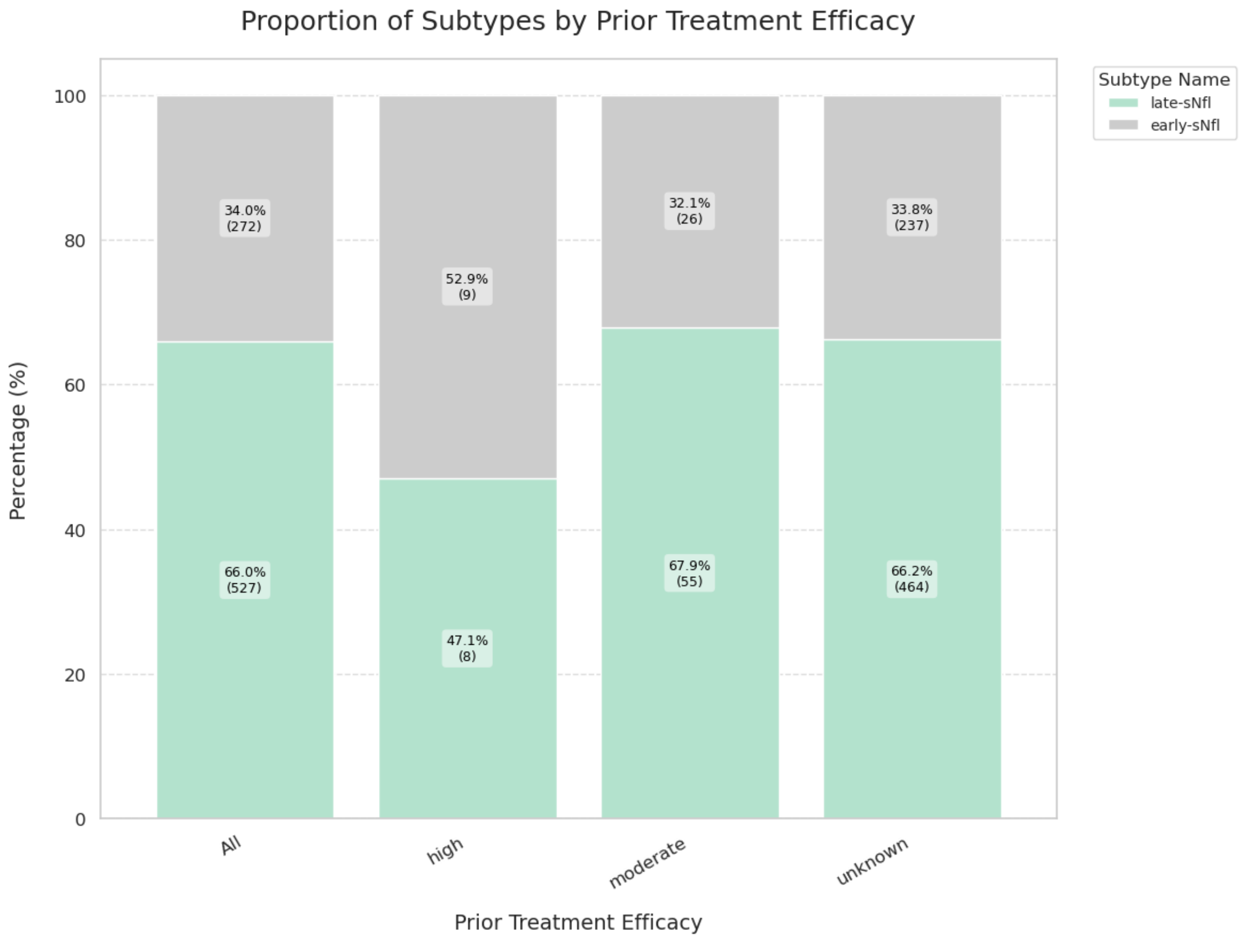

Caption: The plot shows the overall proportion of Early-sNfL and Late-sNfL subtypes in the training set (All), as well as the proportions stratified by prior treatment efficacy (high, moderate) and for subjects with unknown prior treatment (unknown).

Abbreviations: sNfL, serum Neurofilament Light (chain);

Supplementary Figure 5: Order of progression of abnormality in the three subtypes MRI-sNfL model.

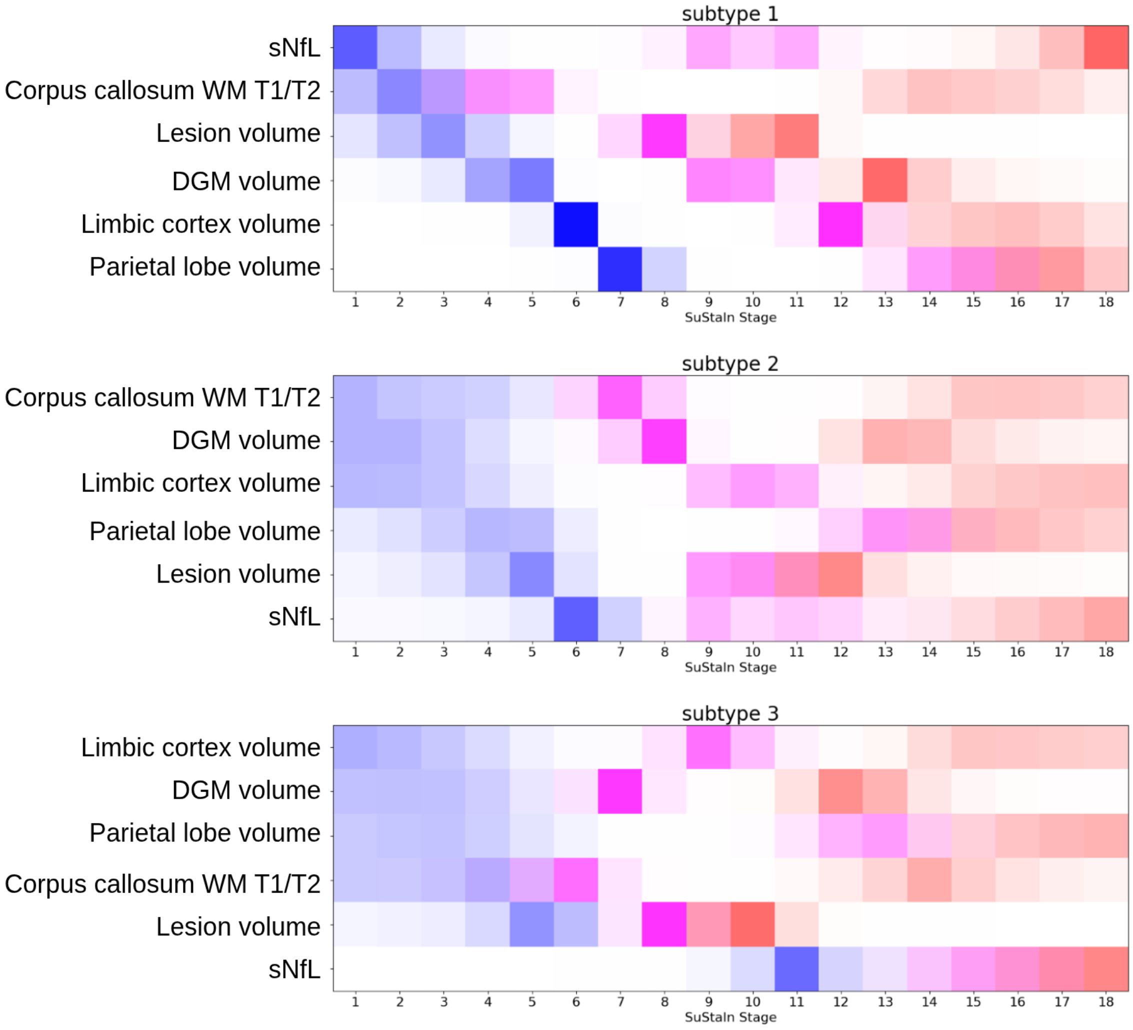

Caption: The positional variance plot, derived from three-subtypes MRI-sNfL model, illustrates the uncertainty in classifying each biomarker (listed vertically) to a specific disease stage (shown horizontally). Three colours represent mild, moderate, and severe abnormalities (corresponding to z-scores of 4, 6, and 8), with shading indicating the model's confidence in the classification at each stage. Subtype 1 is characterised by early sNfL elevation, reduction of the normal-appearing corpus callosal T1/T2 ratio, and lesion accrual. Subtypes 2 and 3 display highly similar patterns, with early volume loss and corpus callosal T1/T2 ratio reduction, followed by sNfL elevation and lesion accrual at later stages, suggesting redundancy between these subtypes.

Abbreviations: sNfL, serum Neurofilament Light (chain); WM, White Matter; DGM, Deep Grey Matter; SuStaIn, Subtype and Stage Inference;

Supplementary tables

Supplementary Table 1 Brain regions from tissue segmentation.

Deep Gray Matter	Brain Stem	Cerebellum
Frontal Lobe	Pons	Cerebellar White Matter
Limbic Cortex	White Matter	Lateral Ventricle
Occipital Lobe	Temporal White Matter	Ventricle
Parietal Cortex	Cingulate White Matter	Optic Chiasm
Temporal Lobe	Corpus Callosum White Matter	

Supplementary Table 2 MRI-derived variables used in SuStaIn models. Variables in bold were utilised in developing the novel SuStaIn model, while underlined variables were employed in the pre-trained model from Eshaghi et al. (2021). The ten variables most highly correlated with EDSS are marked with an asterix *.

**<u>Deep Gray Matter volume*</u>**	Brain Stem volume	Cerebellum volume
<u>Frontal Lobe volume*</u>	Pons volume	Cerebellar White Matter volume
**<u>Limbic Cortex volume*</u>**	White Matter volume	Lateral Ventricle volume
<u>Occipital Lobe volume*</u>	Temporal White Matter volume	Ventricle volume
**<u>Parietal Cortex volume*</u>**	Cingulate White Matter volume	Optic Chiasm volume
<u>Temporal Lobe volume*</u>	Corpus Callosum White Matter volume	**<u>Lesion volume*</u>**
Deep Gray Matter T1/T2	Brain Stem T1/T2	Cerebellum T1/T2
Frontal Lobe T1/T2*	Brain Stem Pons T1/T2	<u>Cerebellar White Matter T1/T2*</u>
Limbic Cortex T1/T2	White Matter T1/T2	Lateral Ventricle T1/T2
Occipital Lobe T1/T2	<u>Temporal White Matter T1/T2</u>	Ventricle T1/T2
<u>Parietal Cortex T1/T2</u>	<u>Cingulate White Matter T1/T2</u>	Optic Chiasm T1/T2
<u>Temporal Lobe T1/T2</u>	**<u>Corpus Callosum White Matter T1/T2*</u>**	

Supplementary Table 3 Demographic and clinical description of the results from the MRI model with 13 variables.

	Training			External Test		
	Lesion-led	GM-led	NAWM-led	Lesion-led	GM-led	NAWM-led
% sample	119 (56%)	60 (28%)	34 (16%)	221 (50%)	152 (34%)	72 (16%)
% of females in each subtype	79 (66%)	54 (90%)	15 (44%)	136 (62%)	127 (84%)	23 (32%)
Age	44 ± 10	41 ± 9	35 ± 10	32 ± 8	31 ± 8	28 ± 8
MS types	RRMS = 94 (79%)<br>SPMS = 25 (21%)	RRMS = 58 (97%)<br>SPMS = 2 (3%)	RRMS = 32 (94%)<br>SPMS = 2 (6%)	Early MS = 221 (100%)	Early MS = 152 (100%)	Early MS = 72 (100%)
Median EDSS	3.5	3.0	2.0	1.5	1.5	1.5

Supplementary Table 4 Demographic and clinical description of the results from the 5-MRI model.

	Training		External Test	
	MRI subtype 1	MRI subtype 2	MRI subtype 1	MRI subtype 2
% sample	142 (75%)	47 (25%)	293 (66%)	147 (33%)
% of females in each subtype	117 (82%)	14 (30%)	216 (74%)	66 (45%)
Age	42 ± 10	41 ± 12	32 ± 8	30 ± 8
MS types	RRMS = 124 (87%)<br>SPMS = 18 (13%)	RRMS = 37 (79%)<br>SPMS = 10 (21%)	Early MS = 293 (100%)	Early MS = 147 (100%)
Median EDSS	3.0	3.5	1.5	2.0

Supplementary Table 5 Treatment classification for therapies received prior to recruitment in the study.

Moderate efficacy	High efficacy
• INTERFERON BETA-1A<br>• INTERFERON BETA-1B<br>• FINGOLIMOD<br>• GLATIRAMER ACETATE	• CLADRIBINE<br>• MITOXANTRONE<br>• DACLIZUMAB<br>• OFATUMUMAB<br>• NATALIZUMAB

Supplementary Table 6 Linear mixed effect models for the annual change of active lesion counts on each subtype and treatment arm.

Subtype	Treatment groups	Training *Coefficient (95% CI)*	*p*	External Test *Coefficient (95% CI)*	*p*
**Lesion-led**	**Both**	-1.961 [-3.277, -0.645]	**0.003**	0.076 [-0.101, 0.252]	0.402
	**Control**	0.366 [-0.833, 1.565]	0.550	0.688 [-0.082, 1.457]	0.080
	**Treatment**	-3.970 [-6.143, -1.798]	**< 0.001**	0.051 [-0.132, 0.234]	0.584
**GM-led**	**Both**	-0.684 [-2.660, 1.292]	0.498	0.133 [0.052, 0.214]	**0.001**
	**Control**	-0.477 [-4.155, 3.200]	0.799	0.286 [0.049, 0.522]	**0.018**
	**Treatment**	-0.867 [-1.765, 0.030]	0.058	0.121 [0.033, 0.209]	**0.007**
**NAWM-led**	**Both**	-0.149 [-1.108, 0.810]	0.761	0.177 [0.004, 0.349]	**0.045**
	**Control**	1.262 [-0.151, 2.675]	0.08	0.300 [-0.320, 0.920]	0.343
	**Treatment**	-1.744 [-2.752, -0.737]	**0.001**	0.166 [-0.018, 0.349]	0.077

Supplementary Table 7 Cox regression analysis for SuStaIn subtypes on the risk of developing new lesions based on treatment group.

**Survival Analysis on "time to radiological disease activity" (new gad-enhancing lesions)**		
**Treatment-adjusted Hazard Ratio [95% CIs] (p-value) for Lesion-led and NAWM-led compared to GM-led (Reference Group)**		
	**Training set**	**External test set**
NAWM-led	1.73 [0.75, 4.01] (*p* = 0.20)	1.10 [0.73, 1.66] (*p* = 0.65)
Lesion-led	1.25 [0.64, 2.46] (*p* = 0.52)	1.02 [0.75, 1.39] (*p* = 0.90)
**Subtype**	**Hazard Ratio [95% CIs] (p-value) for Treated subjects compared to Controls (Reference Group)**	
	**Training set**	**External test set**
Lesion-led	0.10 [0.03, 0.32] (p < 0.001)	0.44 [0.28, 0.69] (p < 0.001)
GM-led	0.22 [0.05, 1.02] (p = 0.053)	0.34 [0.20, 0.59] (p < 0.001)
NAWM-led	0.11 [0.01, 0.84] (p = 0.034)	0.37 [0.17, 0.80] (p = 0.012)

Cox regression analysis results within subtypes of the MRI-only model, showing the risk of developing new gadolinium-enhancing lesions based on assigned treatment groups. Hazard ratios are reported with 95% confidence intervals and p-values.

Supplementary Table 8 Correlation between MRI-sNfL model stages and demographic/clinical variables, with and without ComBat harmonisation.

	**Spearman correlation with MRI-sNfL model stages**					
	**With ComBat harmonisation**			**Without ComBat harmonisation**		
	**All**	**Early-sNfL**	**Late sNfL**	**All**	**Early-sNfL**	**Late sNfL**
**Age**	0.031 (p = 0.517)	0.125 (p = 0.041)	0.126 (p = 0.102)	0.032 (p = 0.502)	0.012 (p = 0.829)	0.122 (p = 0.215)
**EDSS**	0.163 (p < 0.001)	0.196 (p = 0.001)	0.075 (p = 0.336)	0.155 (p = 0.001)	0.173 (p = 0.002)	0.063 (p = 0.522)
**Number of Active Lesions**	0.636 (p < 0.001)	0.571 (p < 0.001)	0.564 (p < 0.001)	0.528 (p < 0.001)	0.651 (p < 0.001)	0.274 (p = 0.005)

Supplementary Table 9 Correlations of demographic and clinical variables with data-derived stages from our proposed MRI-sNfL model (5 MRI variables) and the MRI-sNfL model using 13 MRI variables from Eshaghi et al.

	Training			External Test		
	**Spearman correlation with model stages from the proposed MRI-sNfL model**					
	**All**	**Early-sNfL**	**Late sNfL**	**All**	**Early-sNfL**	**Late sNfL**
**Age**	0.347 (p < 0.001)	0.361 (p = 0.002)	0.343 (p < 0.001)	0.031 (p = 0.517)	0.125 (p = 0.041)	0.126 (p = 0.102)
**EDSS**	0.420 (p < 0.001)	0.396 (p < 0.001)	0.457 (p < 0.001)	0.163 (p < 0.001)	0.196 (p = 0.001)	0.075 (p = 0.336)
**Number of Active Lesions**	0.093 (p = 0.234)	0.115 (p = 0.330)	0.109 (p = 0.301)	0.636 (p < 0.001)	0.571 (p < 0.001)	0.564 (p < 0.001)
	**Spearman correlation with model stages from the MRI-sNfL model using 13 MRI variables**					
	**All**	**Early-sNfL**	**Late sNfL**	**All**	**Early-sNfL**	**Late sNfL**
**Age**	0.125 (p = 0.109)	0.079 (p = 0.446)	0.240 (p = 0.042)	0.082 (p = 0.087)	0.066 (p = 0.417)	-0.066 (p = 0.268)
**EDSS**	0.169 (p = 0.029)	0.072 (p = 0.486)	0.344 (p = 0.003)	0.088 (p = 0.065)	-0.003 (p = 0.973)	0.128 (p = 0.030)
**Number of Active Lesions**	0.154 (p = 0.047)	0.171 (p = 0.097)	0.121 (p = 0.312)	0.314 (p < 0.001)	-0.029 (p = 0.726)	0.416 (p < 0.001)

Supplementary methods

Intensity calibration

This section details the calibration process for the T1-weighted/T2-weighted ratio pipeline described in Section 2.2. Intensity calibration of the T1-weighted and the T2-weighted images is a critical intermediate step before computing their ratio. The objective of this step is to normalise T1-weighted and T2-weighted image intensities to a standardised scale, mitigating the significant impact of variations in MR acquisition settings on image intensity scales. Our calibration approach, inspired by Ganzetti et al.,[41] employs a mask that selects voxels from regions either without myelin (non-ventricular CSF, third and lateral ventricles) or less susceptible to myelin alterations due to

MS, such as the skull. Utilising this mask, we identify a high-intensity value (98th percentile) H and a low-intensity value (2nd percentile) L. Subsequently, each intensity X in the image undergoes linear scaling using the formula (X - L) / (H - L).

Variables standardisation

To standardise volumetric and T1/T2 ratio variables, we used a reference distribution derived from a cohort of 1,105 healthy individuals in the Human Connectome Project (HCP) study.[39] From this distribution, we calculated the mean and standard deviation for each variable, which were then used to standardise the same variable in both the training and external test datasets. For total lesion volume, where no reference distribution from healthy individuals was available, we adopted an alternative normalization approach. Given its exponential distribution, we fitted an exponential model and used the cumulative distribution function (CDF) to assign each observation a score between 0 and 1. This score was subsequently rescaled to a 0–5 range to match the scales of other variables. The provided sNfL levels were pre-normalized, requiring no additional preprocessing.

Analysing the model using 13 MRI variables from Eshaghi et Al

To evaluate the impact of using a reduced set of MRI variables, we compared the proposed MRI-sNfL model (using 5 MRI variables) with a counterpart trained using the full set of 13 MRI variables from Eshaghi et al[8]. Both models were trained on the same training set, with sNfL included in the biomarkers set, and their output stages were assessed based on correlations with key clinical and biological markers.

Supplementary Results

The results of this comparison are summarised in **Supplementary Table 9**. The proposed MRI-sNfL model demonstrates substantially stronger correlations with EDSS than the 13-variable model, both in the training set (ρ = 0.420 vs. 0.169) and the external test set (ρ = 0.163 vs. 0.088), indicating better alignment with clinical disability. In the training data, the proposed model also captures age-related variation more effectively (ρ = 0.347 vs. 0.125). In external validation, correlations with age are low for both models, though the proposed model performs slightly better. While the 13-variable model shows a higher correlation with the number of active lesions in the training set (ρ = 0.154 vs. 0.093), the proposed model substantially outperforms it in the external test set (ρ = 0.636 vs. 0.314), reflecting improved generalisation in capturing acute inflammatory activity.

Comparison with a conventional clustering method

We compared our proposed MRI-sNfL model with a conventional hierarchical clustering approach to assess whether the added complexity of SuStaIn for subtype discovery yields improved predictive performance and interpretability compared to traditional machine learning methods. As a benchmark, we implemented standard hierarchical clustering using the agglomerative (bottom-

up) method to derive two clusters based on the same six features used in our model, all extracted at the screening visit. These features were z-scored, as described in the Supplementary Section "Variable standardisation." Agglomerative clustering begins by treating each subject as its own cluster and iteratively merges the closest pairs based on a defined linkage criterion. We used Ward's linkage, a commonly adopted method that minimizes total within-cluster variance. This process continues until the desired number of clusters—in this case, two—is obtained. We used the implementation of this algorithm provided in scikit-learn (version 1.5.1). In terms of interpretability, SuStaIn provides positional variance diagrams (see **Figure 2**) that capture the sequence and uncertainty of abnormal events within each subtype. In contrast, clusters derived from agglomerative clustering can only be interpreted through population-level summary statistics. For predictive performance, we repeated the survival analysis described in the Methods section and evaluated the two resulting clusters by analysing the risk of new lesion development, adjusting for treatment effects.

Supplementary Results

Cluster assignment based on agglomerative clustering was not significantly associated with the risk of developing new lesions in either the training set (HR = 1.33, 95% CI = [0.71, 2.50], p = 0.38) or the external test set (HR = 1.19, 95% CI = [0.92, 1.63], p = 0.24). In contrast, subtypes identified by our proposed MRI-sNfL model showed a significant difference in the training set, with similar—though not statistically significant—trends observed in the external test set (see Results section).
